# Duty of Medical Men

**Published:** 1853-06

**Authors:** 


From the Medical Times and Gazette, March 26. 1853.

*Duty of Medical Men.*

Dr. Todd, in his farewell address, on resigning his professorship, made the following remarks:—

"It appears to me, that when a man proposes to devote himself to the practice of an honorable profession, he has a twofold duty to perform; first, to fit himself to the utmost of his ability for the practical duties of that profession; and, secondly, having done so, it is incumbent on him to divest himself, as far as possible, of every engagement which may interfere with his bending his thoughts and attention to the various anxious, difficult, and often perplexing questions which are continually arising in the course of his professional practice. Every member of a liberal profession should keep it constantly in view that he exercises his calling not simply for his own personal benefit, but for the public good, and for the good of his profession at large. So every practising physician or surgeon, whether the sphere of his labor be within wide or narrow limits, should bear in mind that in the successful application of his art, by fair, honorable, and truthful means, is involved the repute and estimation in which his profession is held by the public at large. Let each of us act under the feeling, that to himself specially is committed the keeping of the honorable character and the scientific credit of our common profession, and he will have the strongest motives, not only to eschew everything that savors of charlatanical pretence, but to seek for and insure the highest means of moral and intellectual culture."

